# Treatment of unilateral spatial neglect after stroke using transcranial direct current stimulation (ELETRON trial): study protocol for a randomized controlled trial

**DOI:** 10.1186/s13063-016-1598-4

**Published:** 2016-10-03

**Authors:** Gustavo José Luvizutto, Gabriela Rizzo Soares Rizzati, Marcelo Ortolani Fogaroli, Rodrigo Thomazi Rodrigues, Priscila Watson Ribeiro, Hélio Rubens de Carvalho Nunes, Gabriel Pereira Braga, Rafael Dalle Molle da Costa, Silméia Garcia Zanati Bazan, Luiz Antônio de Lima Resende, Adriana Bastos Conforto, Rodrigo Bazan

**Affiliations:** 1 Rehabilitation Department, Botucatu Medical School, Professor Montenegro Ave., Botucatu, Brazil; 2 Botucatu Medical School, Professor Montenegro Ave., Botucatu, Brazil; 3 Department of Neurology, Psychology and Psychiatry, Botucatu Medical School, Professor Montenegro Ave., Botucatu, Brazil; 4 Department of Internal Medicine, Botucatu Medical School, Professor Montenegro Ave., Botucatu, Brazil; 5 Neurology Clinical Division, São Paulo University, São Paulo, Brazil; 6 Botucatu School of Medicine, University Estadual Paulista Júlio de Mesquita Filho, District of Rubião Junior, Botucatu, SP 18618-970 Brazil

**Keywords:** Stroke, Unilateral spatial neglect, Transcranial direct current stimulation

## Abstract

**Background:**

Unilateral spatial neglect (USN) is characterized by the inability to report or respond to people or objects that are presented in the spatial hemisphere that is contralateral to the lesioned hemisphere of the brain. USN has been associated with poor functional outcomes and long stays in hospitals and rehabilitation centers. Noninvasive brain stimulation, such as transcranial direct current stimulation (tDCS), has been used in people who have been affected by USN after stroke. The effects of such treatment could provide new insights for health professionals and policy-makers. The aim of this study will be to evaluate the effectiveness and safety of tDCS for USN after stroke.

**Methods:**

A prospective randomized controlled trial with two parallel groups will be conducted, which will aim to recruit 60 patients with USN after ischemic or hemorrhagic stroke. Participants will be randomly placed into the following four treatment groups: (1) anodal tDCS over the right parietal lobe (*n* = 15), (2) cathodal tDCS over the left parietal lobe (*n* = 15), (3) a sham group of anodal tDCS over the right parietal lobe (*n* = 15), and (4) a sham group of cathodal tDCS over the left parietal lobe (*n* = 15). Blinded assessors will conduct two baseline assessments and one post-intervention assessment. The primary outcome measure will be the level of USN as assessed by the conventional Behavioral Inattention Tasks and the Catherine Bergego Scale. Secondary measures will include neurological capacity (based on the Scandinavian Stroke Scale), functional capacity (based on the Functional Independence Measure and Modified Rankin Scale), autonomy (based on the Barthel Index), and quality of life (based on the EuroQol-5D). Group allocation will be concealed, and all analyses will be based on an intention-to-treat principle.

**Discussion:**

This study will explore the effects of more than 15 sessions of tDCS on the level of USN, functional capacity, autonomy, and quality of life in patients with USN after stroke. This proposed study has the potential to identify a new, evidence-based intervention that can enhance perception and independent living in patients with USN after stroke.

**Trial registration:**

REBEC - RBR-78jvzx, registered on 13 March 2016.

## Background

Unilateral spatial neglect (USN) clinically manifests when an individual does not respond to any tactile or visual stimulus on one side of the body or hemispherical space. Such unresponsiveness cannot be attributed to a sensory deficit or a primary motor deficit. This condition makes it difficult for a patient to report, respond to, guide, or interpret any stimulus that is received from the affected side [1–3]. Often a USN is associated with lesions in the right hemisphere of the brain, particularly in the parietal lobe [4–7], or the right posterior parietal lobe [8, 9]. USN is associated with a poor prognosis and the need for long periods of hospitalization [10–14].

Currently, the procedures that are used to evaluate USN are composed of tasks that use pen and paper to distinguish cancellation targets or simultaneous touch [15, 16]. Four tests have been proposed to assess USN, namely, the Face-Hand Test (FHT), which is used to promote simultaneous dual sensory stimulation in individuals [17–19]; the Line Cancellation Task [20, 21] or Star Cancellation [22], both of which are used to grade the severity of the USN based on the number of lines or stars cancelled; and the Line Bisection Task [23, 24] which is used to observe deviation from the midline of a space. The cancellation and bisection tests are used consistently in the clinic and exhibit high sensitivity and specificity for detecting USN [25, 26]. In addition to the cancellation and bisection tests, Azouvi et al. (2003) developed a specific scale based on 10 activities of daily living that can be changed by USN. This scale is typically used during the chronic period after a stroke to measure the efficacy of the treatments for symptom regression [27].

Since the early 1970s, various rehabilitation techniques have been proposed to reduce the disability that is caused by USN after stroke. Among them include techniques for noninvasive neuromodulation through brain stimulation, such as, transcranial magnetic stimulation (rTMS) and transcranial direct current stimulation (tDCS), which create low-intensity electrical currents in the brain to change the excitability of cortical regions [28, 29]. Recent studies suggest that noninvasive brain stimulation techniques can aid in the rehabilitation of patients with stroke to promote the recovery of function [30–33].

tDCS reversibly polarizes regions of the brain via the topical application of low-intensity direct currents to change the potential and modulation of transneuronal membrane excitability levels and firing rates [31, 34]. The tDCS-induced polarization effect in the brain varies depending on electrode polarity, wherein the anodal stimulation (positive electrode) increases cortical excitability, while the cathodal stimulation (negative electrode) decreases excitability [35]. Recent human studies have shown that anodal polarization increases the excitability of the motor, visual, and prefrontal cortices, with an improved performance of motor skills [36], working memory [37], and verbal fluency [38].

Few studies have been designed to evaluate the effect of tDCS on the USN syndrome after stroke. A randomized controlled trial conducted by Ko et al. (2008) in 15 patients with USN after stroke observed improvements in the performance of the Line Cancellation Task, Figure and Shape Copying, and the Line Bisection Task after anodal polarization using tDCS of the right posterior parietal region [39]. Using tDCS in 10 patients with USN after stroke, Sparing et al. (2009) observed that inhibitory cathodal stimulation applied to the uninjured posterior parietal cortex, and excitatory anodal stimulation of the lesioned posterior parietal cortex, reduced the degree of USN [40].

In a clinical trial conducted in 10 patients with USN after stroke in the chronic phase, Sunwoo et al. (2013) evaluated the following three types of stimulation: (1) simultaneous anodal stimulation to the right parietal cortex and cathodal stimulation to the left parietal cortex, (2) single-mode anodal stimulation to the right parietal cortex, and (3) sham stimulation to control for the placebo effect. The authors observed an improvement in the performance of the Line Bisection Tasks for the simultaneous and single-mode stimulation types. Moreover, the effect for simultaneous stimulation was greater than that compared to the other two stimulation types [41]. A recent study evaluated the application of tDCS in five sessions, each of 20 min, in five individuals with USN in the chronic phase of stroke. Cathodal stimulation was applied to the P3 region (left parietal cortex) and anodal stimulation was applied to the P4 region (right parietal cortex) according to the international electroencephalogram system. The results were not statistically significant and the degree of USN was not reduced at the end of treatment. These results suggest that randomized controlled trials should include the highest possible number of patients [42].

USN after stroke can be caused by a hemispherical imbalance of cerebral electrical activity with the injured area showing no decrease in cortical excitability. Recent studies suggest that spatial perception could be improved by restoring the balance of hemispherical activity through tDCS [40–43]. In addition, USN has been associated with a worse functional outcome, a longer retention of patients in rehabilitation centers, a high risk of falls, and the need for a wheelchair. Such outcomes decrease productivity and quality of life compared to other patients without USN after stroke [13, 14, 43].

Several studies have reported that tDCS can improve performance in tasks that assess spatial orientation after USN such as Line Bisection and Line Cancellation Tasks. However, these studies do not report a complementary reduction in long-term disability or an improvement in patient quality of life. Because tDCS is a cost-effective noninvasive procedure for brain stimulation compared to, for example, magnetic stimulation, we propose the present research to try to establish whether it could in fact lead to the aforementioned quality of life improvements.

### Aims

The principal objective of this study in patients with USN after stroke is to evaluate the efficacy and safety of 15 sessions of tDCS versus placebo in ameliorating USN. We hypothesize that tDCS will induce changes in the neuronal activity of the posterior parietal lobe that will reduce USN presentation in patients after stroke.

In addition, we will assess the effects of tDCS on the capacity of function, level of paralysis, and quality of life.

## Methods

### Design

**Fig. 1:**
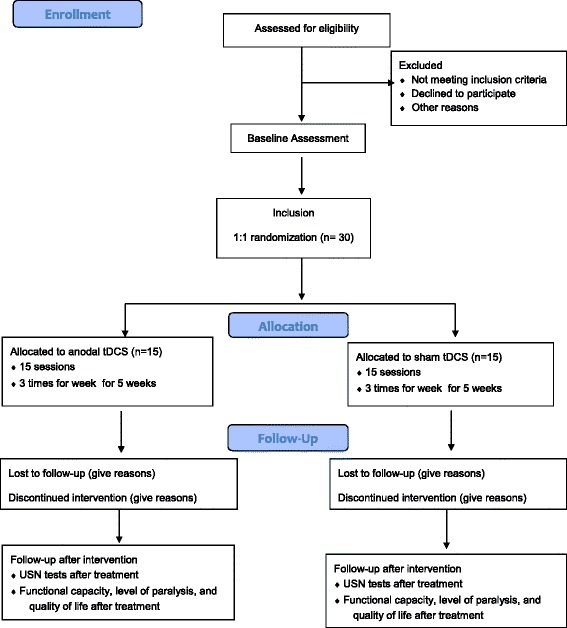
Study flow diagram for anodal transcranial direct current stimulation (tDCS) stimulation

**Fig. 2:**
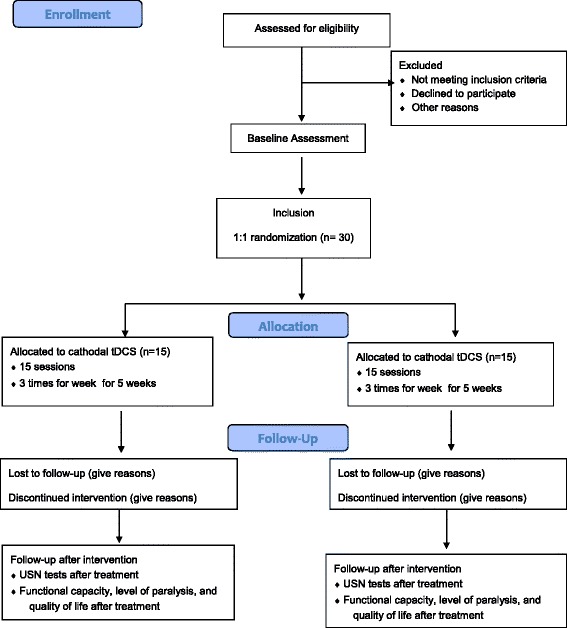
Study flow diagram for cathodal transcranial direct current stimulation (tDCS) stimulation

This single-center, randomized, placebo-controlled, double-blind, parallel-group study of 60 patients with USN will be conducted in accordance with the Consolidated Standards of Reporting Trials (CONSORT) 2010 flow diagram [44] (Figs. 1 and 2).

### Sample size

We estimate a minimum sample size per group of 15 assuming: simple random sampling; type I and II error probabilities equal 0.05 and 0.20, respectively; the absence of confounders; normal distribution for the outcome “percentage of deviation” with a mean baseline equal to a 15 % reduction in the outcome after tDCS; an estimated reduction of between 8 and 3 % for the sham group; and a variation coefficient of 20 %.

### Location and setting

All assessments will be conducted at the Botucatu Medical School within the Department of Neurology and Center of Rehabilitation, Botucatu, Brazil.

## Participants

### Inclusion criteria

Individuals of 18–85 years of age and of either sex, with USN after stroke diagnosis within 15 weeks of the start of stroke symptoms, will be included in this study. The patients will be recruited in the stroke unit by Botucatu Medical School, and will include patients with right-hand ischemic or hemorrhagic stroke in the right parietal lobe confirmed using computed tomography (CT) or magnetic resonance imaging (MRI). USN will be objectively diagnosed by conventional Behavior Inattention Tasks (BIT-C) with a cutoff of >129.

### Exclusion criteria

Excluded individuals will include any patients with metal-in-cranium injuries near the electrode placement area, a cardiac pacemaker in situ, intracerebral vascular clips or any other electrically sensitive support system, clinical instability, epilepsy, severe cognitive impairment, bilateral lesions, global aphasia, previous visual disturbances, depression with scores >8 in the Hospital Anxiety and Depression Scale (HAD), pregnancy, or other neurological diseases.

### Procedures (template)

Individuals diagnosed with right-hand stroke after discharge from the hospital will be sent to the rehabilitation sector. All individuals with damage to the right hemisphere, as confirmed by a CT or MRI scan, will be invited to participate. The patient, a family member, or guardian must sign a Free and Informed Consent Form. After signing the Consent Form, the individuals will be evaluated to confirm their diagnosis using BIT-C, consisting of Star Cancellation (SC), Letter Cancellation (LC), Line Crossing (LiC), Line Bisection (LB), Figure and Shape Copying (FSC-A&B), and Representational Drawing (RD). After confirmation of the USN diagnosis (BIT-C >129), patients will be screened to clinical condition, the HAD, the Edinburgh Handedness Inventory to classify laterality, and checking of the excluded criteria for the tDCS application. After screening patients will be randomized into the following four groups: (1) treatment with anodal tDCS, (2) control with anodal tDCS (sham mode), (3) treatment with cathodal tDCS, (4) control with cathodal tDCS (sham mode). Individuals will be assessed using the Catherine Bergego Scale (CBS), the Mini-mental State Examination (MMSE), the National Institutes of Health Stroke Scale (NIHSS), the Functional Independence Measure (FIM), the Barthel Index, the Modified Rankin Scale (mRS), and the European Quality of Life Scale (EuroQol-5D) by an investigator who is blinded to the treatment that the patient received before the first session and after 1 week from the last tDCS session (Table 1).

**Table 1:** Template of recommended content for the schedule of enrollment, interventions, and assessments

	Study period				
	Enrollment	Allocation	Post allocation		Close-out
Timepoint	−t_1_	0	t_1_	t_15_	t_16_
Enrollment:					
Eligibility screen	X				
Informed consent	X				
BIT-C and HAD^a^	X				
MMSE^b^	X				
EHI^c^	X				
Allocation		X			
Interventions:					
Anodal tDCS^d^			X	X	
Cathodal tDCS^e^			X	X	
Anodal placebo			X	X	
Cathodal placebo			X	X	
Assessments:					
BIT-C^a^					X
CBS^f^		X			X
NIHSS, FIM and BI^g^		X			X
mRS^h^		X			X
Quality of life^i^		X			X

^a^Conventional Behavior Inattention Tasks and Hospital Anxiety and Depression Scale

^b^Mini-mental State Examination

^c^Edinburgh Handedness Inventory

^d^Anodal tDCS applied over left parietal lobe (P3)

^e^Cathodal tDCS applied over right parietal lobe (P4)

^f^Catherine Bergego Scale

^g^National Institutes of Health Stroke Scale, Functional Independence Measure and Barthel Index

^h^Modified Rankin Scale

^i^Quality of life measured by The European Quality of Life Scale (EuroQol 5D)

### Randomization and blinding

The concealed randomization schedule will be established using a computer-generated random number sequence, and maintained by an offsite investigator who is neither involved with the enrollment nor with the assessment of study participants. A second research assistant will consecutively open consecutively numbered, randomly ordered, opaque envelopes containing the group allocation (in a 1:1 ratio) after the baseline assessment. All the participants will receive rehabilitation along with 45 min of physical therapy three times a week for 5 weeks after tDCS application. The tDCS will be applied three times a week for 5 weeks for a total of 15 sessions, wherein the patient is evaluated before the beginning of the first session and 1 week after the last session by a blinded investigator.

### Intervention

The tDCS will be applied in accordance with the technique used by Sparing et al. [40]. The patient will be placed in a room with minimal external influences (noise, lamps, and electromagnetic waves), and positioned in a sitting posture with their arms and torso supported on a table at 45°. For stimulation of the right and left posterior parietal lobes, after shaving and cleansing the skin with alcohol, the electrodes will be placed in positions P4 and P3 of the 10/20 International System of Electroencephalography. The reference electrodes will be placed on the cranial vertex (Cz). Four groups of patients will receive stimulation as follows: group 1, right anodal stimulation (P4 anode, reference Cz), group 2, sham control for right anodal stimulation (P4 anode, reference Cz), group 3, left cathodal stimulation (P3 cathode, reference Cz), and group 4, sham control for left cathodal stimulation (P3 cathode, reference Cz). For stimulation, sponge surface electrodes (5 cm × 5 cm square) will be soaked in saline. A continuous current of 1 mA in intensity and a resistance of less than 10 kΩ will be applied for 1200 s with ramping up for 30 s and ramping down for 30 s according to international safety guidelines [38]. The tDCS will be applied using an electrical stimulator, namely the battery-powered DC-Stimulator Plus model, NeuroConn®.

A total of 15 sessions will be performed using a schedule of three times a week for 5 weeks, wherein a blinded examiner evaluates the patient before the beginning of the first session and 1 week after the last session. For the control group (sham), patients will be placed in the same room and position, but the current that will be applied to each control group will be shut off after 30 s.

The physiotherapy protocol will be delivered after tDCS and will be composed of saccadic eye movement training with visual scanning exercises (VSEs) integrated with task-specific activities. The VSEs integrated with task-specific activities will consist of dual-task activities, which require the ability to allocate information-processing resources between two tasks and to maintain sufficient attention on the visual scanning task during the dual-task performance. The guideline of the VSEs integrated with task specific activities and the principles of progression of these exercises follow van Wyk et al. [45], with session durations of approximately 45 min.

### Primary outcome measures

Change in the degree of USN will be measured as follows: (1) D1, as the percentage of changed in range of BIT-C, and (2) D2, based on the CBS.

### Secondary outcome measures

Disability, autonomy, and quality of life will be measured as follows: (1) D3, as the change in neurological status based on the NIHSS, (2) D4, as the change in functional independence based on the Barthel Index, (3) D5, as the change measured using the mRS, and (4) D6, as the change in quality of life based on the EuroQol-5D. For all of the outcomes (except for D6), the change after stimulation will be calculated as follows: 100 (*Vf* − *V0*)/*V0*, where *V0* is the value of the prerandomization variable and *Vf* is the variable value after 1 week of treatment.

### Adverse effects

Any adverse effects will be reported during the study period by the safety questionnaire proposed by Brunoni et al. [46].

### Baseline assessments

#### USN evaluation

*Conventional Behavior Inattention Tasks (BIT-C)*: the six conventional tasks of the BIT-C will be administered, consisting of Star Cancellation (SC), Letter Cancellation (LC), Line Crossing (LiC), Line Bisection (LB), Figure and Shape Copying (FSC-A&B), and Representational Drawing (RD). The BIT-C is usually administered to diagnose USN and provides a range (0–146) and a cutoff score. For all USN tests, the examiner will place the test sheet in front of the patient with a distance of 60 cm between the paper and the glabella of the patient [47].

*Catherine Bergego Scale (CBS)*: this test will be used to measure the extent to which the USN interferes with daily tasks. The scale is divided into 10 activities each with a score of 0 to 3. The maximum scale score is 30 and the total indicates USN severity [27].

#### Neurological and functional evaluation

*Mini-mental State Examination (MMSE)*: this instrument is used for screening cognitive function, and provides measures of orientation, registration (immediate memory), short-term memory (but not long-term memory) as well as language functioning. Scores of 25–30 out of 30 are considered normal; the National Institute for Health and Care Excellence (NICE) classifies 21–24 as mild impairment, 10–20 as moderate impairment, and <10 as severe impairment. The MMSE is used in this trial for data characterization [48].

*Edinburgh Handedness Inventory (EHI)*: the EHI is a measurement scale used to assess the dominance of a person’s right or left hand in everyday activities, sometimes referred to as laterality [49].

*Hospital Anxiety and Depression Scale (HAD)*: the HAD was originally developed by Zigmond and Snaith (1983) and it is used to determine the levels of anxiety and depression that a patient is experiencing, being composed for fourteen items. Seven of the items relate to anxiety and seven relate to depression. Each item on the questionnaire is scored from 0 to 3 which means that a person can score between 0 and 21 for either anxiety or depression [50].

*National Institutes of Health Stroke Scale (NIHSS)*: this test will be used to evaluate the effect of acute cerebral infarction on the levels of consciousness, language, neglect, visual-field loss, extraocular movement, motor strength, ataxia, dysarthria, and sensory loss. The higher the score on this scale the greater the neurological deficit [51].

*Functional Independence Measure (FIM)*: this test will be used to measure the functional independence and autonomy of the subjects in six domains (self-care, sphincter control, mobility, locomotion, communication, and social cognition). The scale scores range from 18 to 126, and the higher the score, the better the autonomy and independence [52].

*Barthel Index*: this test will be used to measure the functional independence and autonomy of the subjects in 10 activities, namely, feeding, bathing, personal care, ability to dress, motility, urinary pace, bathroom use, transfer, mobility, and climbing stairs. The maximum score is 100 which indicates a greater degree of autonomy [51, 53].

*Modified Rankin Scale (mRS)*: this test will be used to evaluate the degree of independence, and determines whether the patient can perform self-care during activities of daily living [51]. The scale is ordinal from 0 to 6, and the greater the score, the worse the functional capacity.

*The European (EuroQol 5D) Quality of Life Scale*; this test will be used to measure the impact of stroke on the subject’s quality of life in five domains, namely, structured mobility, self-care, usual activities, pain/discomfort, and anxiety/depression. The scale scores range from 0 to 10, and the higher the score, the worse the perception of life quality. At the end of the test, patients will mark their health on an ordinal scale from 0 to 100, with a lower score indicating poorer health [54].

### Data analysis

To detect changes in continuous outcomes, a comparison between the anodal tDCS and the cathodal tDCS, as well as between the tDCS and sham stimulation, will be performed using a linear regression model with a normal response. This analysis will be adjusted for potential confounders, such as age, mRS score, and USN score at baseline. This analysis approach does not test the validity of the model’s theoretical assumptions with a normal response. However, if a fault is identified then the comparison will be performed by adjusting the regression model with an asymmetric response. In the latter case, the comparison will be made using the nonparametric Mann-Whitney test. Data will be analyzed using SPSS version 22 (SPSS Inc., Chicago, IL 60606, USA), and will be considered statistically significant at a *p* value < 0.05.

## Discussion

In recent years, tDCS has proven to be a promising tool in neurorehabilitation [52]. Some studies indicate that the application of tDCS over the parietal lobe may be beneficial for treating patients with USN after stroke [39–41]. USN after stroke is associated with a poor functional outcome and long stays in rehabilitation centers. Although various nonpharmacological and pharmacological treatments for USN have been proposed, there is no evidence of efficacy. Thus, studies of tDCS in the treatment of USN may offer new insights for rehabilitation in these patients [10–14].

Patients with USN after stroke have a more difficult rehabilitation process, which is more demanding for the stroke rehabilitation team [12]. The few studies on stroke rehabilitation report interesting results in terms of both USN reduction and modulation of cortical excitability. Nonetheless, these studies report no improvements in patient functional capacity or quality of life. tDCS devices, which are easy to use and portable, could be useful for stroke rehabilitation because their application can modulate the excitability of the parietal cortex after USN [55–57]. Some studies report that anodal stimulation improves USN symptoms in patients after stroke, while others report that cathodal stimulation enhances perceptual performance, or that there is no difference between anodal and cathodal stimulation [40–42]. Thus, we aim to conduct a multicenter randomized controlled trial to investigate whether tDCS has the potential to become a promising treatment for USN after stroke, and to determine the best tDCS type (anodal or cathodal) to improve the symptoms of USN.

This trial, which is one of the multicenter randomized controlled trials to assess the efficacy of tDCS in USN after stroke, may also shed light on whether noninvasive brain stimulation techniques are of value in the comprehensive treatment of USN. We are confident that our study will answer this question and provide strong evidence of the short- and long-term efficacy of modulating parietal lobe excitability via tDCS to treat USN after stroke.

### Trial status

Ongoing.

## Abbreviations

BIT-C: Conventional Behavioral Inattention Tasks
CBS: Catherine Bergego Scale
CT: Computer tomography
EHI: Edinburgh Handedness Inventory
EuroQol: The European (5D) Quality of Life Scale
FSC-A&B: Figure and Shape Copying
HAD: Hospital Anxiety and Depression Scale
LB: Line Bisection
LC: Letter Cancellation
LiC: Line Crossing
MMSE: Mini-mental State Examination
MRI: Magnetic resonance image
mRS: Modified Rankin Scale
NIHSS: National Institutes of Health Stroke Scale
RD: Representational Drawing
rTMS: Repetitive magnetic stimulation
SC: Star Cancellation
tDCS: Transcranial direct current stimulation
USN: Unilateral spatial neglect
